# Plant-mediated synthesis of silver nanoparticles: unlocking their pharmacological potential–a comprehensive review

**DOI:** 10.3389/fbioe.2023.1324805

**Published:** 2024-01-09

**Authors:** Rajan Dhir, Sakshi Chauhan, Praddiuman Subham, Saksham Kumar, Pratham Sharma, Amrullah Shidiki, Gaurav Kumar

**Affiliations:** ^1^ Department of Microbiology, School of Bioengineering and Biosciences, Lovely Professional University, Punjab, India; ^2^ Department of Microbiology, National Medical College and Teaching Hospital, Birgunj, Nepal

**Keywords:** silver nanoparticles, medical application, pharmaceutical, antibacterial, cytotoxic

## Abstract

In recent times, nanoparticles have experienced a significant upsurge in popularity, primarily owing to their minute size and their remarkable ability to modify physical, chemical, and biological properties. This burgeoning interest can be attributed to the expanding array of biomedical applications where nanoparticles find utility. These nanoparticles, typically ranging in size from 10 to 100 nm, exhibit diverse shapes, such as spherical, discoidal, and cylindrical configurations. These variations are not solely influenced by the manufacturing processes but are also intricately linked to interactions with surrounding stabilizing agents and initiators. Nanoparticles can be synthesized through physical or chemical methods, yet the biological approach emerges as the most sustainable and eco-friendly alternative among the three. Among the various nanoparticle types, silver nanoparticles have emerged as the most encountered and widely utilized due to their exceptional properties. What makes the synthesis of silver nanoparticles even more appealing is the application of plant-derived sources as reducing agents. This approach not only proves to be cost-effective but also significantly reduces the synthesis time. Notably, silver nanoparticles produced through plant-mediated processes have garnered considerable attention in recent years due to their notable medicinal capabilities. This comprehensive review primarily delves into the diverse medicinal attributes of silver nanoparticles synthesized using plant-mediated techniques. Encompassing antimicrobial properties, cytotoxicity, wound healing, larvicidal effects, anti-angiogenesis activity, antioxidant potential, and antiplasmodial activity, the paper extensively covers these multifaceted roles. Additionally, an endeavor is made to provide an elucidated summary of the operational mechanisms underlying the pharmacological actions of silver nanoparticles.

## Graphical Abstract

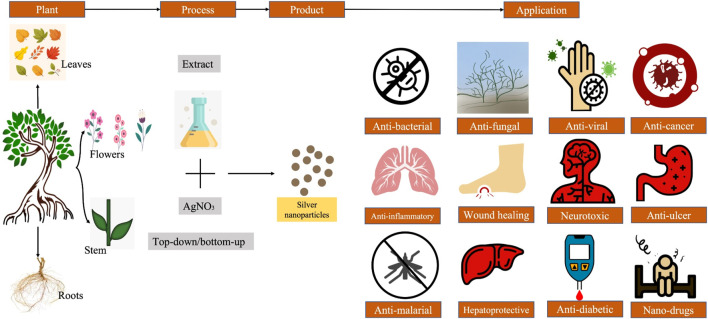

## 1 Introduction

In 1959, physicist Richard Feynman laid the groundwork for a groundbreaking concept—the manipulation and precise control of individual atoms and molecules. It was not until a decade later, in 1960, that Norio Taniguchi coined the term “nanotechnology” to encapsulate this transformative vision. However, the journey toward what we now recognize as modern nanotechnology began to take shape in 1981 with the invention of the scanning tunneling microscope (Hulla et al., 2015). This momentous advancement marked the inception of a scientific revolution, enabling the manipulation of matter at the atomic and molecular scale. Nanotechnology has rapidly surged in prominence and now extends its influence across diverse domains, including healthcare, cosmetics, biomedicine, food production, gene delivery, environmental preservation, mechanics, optics, chemical industries, space exploration, energy science, nonlinear optical devices, and photoelectrochemical applications (Ahmed et al., 2016b). Notably, the fusion of nanotechnology with medical practice has given birth to an enthralling field—nanomedicine, which has become the focal point of extensive research and garnered considerable attention among scholars (Ghavanloo et al., 2023). The collective efforts in the realm of nanomedicine have yielded novel nanomaterials, with nanoparticles at the forefront, and innovative nanotherapeutics. Furthermore, this pursuit has catalyzed advancements in diagnostic methodologies, contrast agents, and the development of cutting-edge medical devices. This dynamic landscape is reshaping the future of medicine, enhancing our capacity to diagnose, treat, and improve human health.

Nanoparticles, sized from 1 to 100 nm, display diverse shapes–spheres, rods, cubes, tubes, and intricate structures–each contributing unique traits. Maintaining precise control is essential to handle size variability within samples. Spherical nanoparticles act as a reliable reference due to their symmetrical structure. Nanorods, with adjustable aspect ratios, influence optical and electrical characteristics. Well-defined geometric shapes of nanocubes and cuboids significantly impact reactivity. Elongated nanotubes and nanowires find applications in electronics. Two-dimensional nanoplates and nanosheets substantially affect the surface area and reactivity. Shapes like stars or polyhedra possess distinctive characteristics. Regardless of shape, nanoparticles, with a significant surface area, play a crucial role in medicine, catalysis, electronics, and materials research due to their impactful reactivity, as well as optical, electrical, and magnetic properties. In recent years, metal nanoparticles, particularly silver nanoparticles (AgNPs or SNPs), have gained considerable attention for their multifaceted applications in the field of medicine. AgNPs have risen to prominence, primarily owing to the innate antibacterial properties of bulk silver, setting them apart as a noteworthy category among the various nanoparticles that have been meticulously explored and characterized. Their remarkable attributes are intrinsically tied to their physical dimensions, encompassing size, shape, and morphology, which endow them with unique functionalities. This distinctive quality enables silver nanoparticles to engage with a wide spectrum of biological organisms, spanning from plants and animals to microbes (Luceri et al., 2023). The past few years have witnessed a surge in dedicated research and investigation into the potential applications of AgNPs, signalling a promising future in the realm of science. Beyond their well-documented antimicrobial efficacy against a diverse range of microscopic pathogens, AgNPs have unveiled their potential across an array of scientific domains. These applications encompass revolutionary advances in wound healing, innovative strategies for retinal therapies, and the development of pharmaceutical solutions, including their role in anticancer and antioxidant therapies. Beyond these healthcare applications, AgNPs continue to exhibit substantial relevance in conventional domains such as electronics, catalysis, and Raman scattering. As the exploration of AgNPs progresses, their diverse and evolving roles within the scientific landscape promise to revolutionize various fields, enhancing both healthcare and technology.

These AgNPs can be manufactured using chemistry, physics, or through the use of green chemistry, an approach that is more kind to the environment. The biogenic production of nanoparticles is an important aspect of green chemistry, and it can be accomplished with the help of fungus, yeast, bacteria, actinomycetes, and plant extracts. Plant-mediated production of nanoparticles can be accomplished with the help of components of plants such as the leaves, stems, and roots (Olga et al., 2022). In the field of chemistry, the term “green chemistry” refers to practices that aim to reduce the number of harmful chemicals that are released into the environment while also reducing the amount of trash that is produced. It is favored over the more traditional method of chemical synthesis because the latter requires the reduction of a variety of metals through the use of dangerous chemicals. Researchers have developed a thirst for ever-evolving procedures or techniques of green chemistry for the synthesis of metal nanoparticles, which has caused them to switch to environmentally friendly techniques to generate well-characterized nanoparticles. This has led to an increase in the use of green chemistry in the production of nanoparticles. In addition, minimizing waste by utilizing green nanocatalyst production and creating unique green catalytic approaches that can humorously improve reaction selectivity and overall process efficiency are both important steps (Kharissova et al., 2019). The purpose of this article is to offer a concise summary of the procedures that are utilized in the production of silver nanoparticles, in addition to their characterization and the possible therapeutic applications of these particles.

The biological impacts of AgNPs, including antibacterial characteristics, cytotoxicity, magnetic behavior, and redox potential, are intricately associated with their shape and size, as delineated in multiple studies (Cheon et al., 2019; Khodashenas and Ghorbani, 2019). Altering surface attributes, configurations, and dimensions can impact these characteristics and their method of absorption, contributing to biocompatibility (Ferdous & Nemmar, 2020). Zhang et al. (2016) emphasized the effectiveness of positively coated NPs as carriers for drug delivery, implying that adjusting the charge influences their drug delivery potential. Other attributes, like antimicrobial activity, are similarly contingent on shape and size; at 55 nm, sphere, and wire AgNPs exhibit lower bactericidal potential than nanocubes (Hong et al., 2016), while triangular AgNPs showcase robust biocidal activity compared to cube and rod AgNPs, indicating shape and size-dependent interactions with *Escherichia coli* (Pal et al., 2007). Conversely, spherical, triangular, and cuboid AgNPs exhibit no discernible effect on *Staphylococcus aureus* (Actis et al., 2015). In a recent investigation by Cheon et al. (2019), the antimicrobial efficacy of differently shaped AgNPs varied based on total surface area and the released amount of Ag ions against *E. coli, S. aureus,* and *Pseudomonas aeruginosa*. Spherically shaped AgNPs demonstrated superior effectiveness, forming a comparatively larger inhibition zone than disk and triangular shapes. Another study by Kumari et al. (2017) entailed the synthesis of nanoparticles with diverse shapes, including spherical (2–5 nm and 40–50 nm), rectangular (45–65 nm), and pentagonal/hexagonal (50–100 nm). Antimicrobial assessments against *Escherichia coli, Staphylococcus marcescens, P. aeruginosa,* and *Shigella sonnei* revealed that small spherical NPs displayed the highest activity, credited to their greater surface area, while the sharp edges of pentagonal and hexagonal NPs were posited as potential factors contributing to their activity.

## 2 Synthesis of silver nanoparticles using plant extracts

The synthesis of AgNPs can generally be accomplished using either the bottom-up or the top-down approach. Top-down includes slicing or cutting down a complex entity (bulk material) into its nano-sized counterparts, whereas bottom-up refers to the process of building up material from atoms to molecules to clusters (Husen and Siddiqi, 2014). The preparation of AgNPs typically takes place in a homogenous system during the bottom-up approach. This is because the bottom-up strategy utilizes a catalyst to synthesize nanostructures, which the catalyst then controls. In general, the top-down process includes the reduction in size of the material, which is accomplished through the use of specialized ablations such as cutting, thermal decomposition, etching, mechanical grinding, and sputtering. The top-down methodology suffers from the most significant structural flaw known as an ace defect. The physical characteristics and surface chemistry of silver nanoparticles are both substantially altered as a result of these defects (Siddiqi et al., 2018).

**FIGURE 1:**
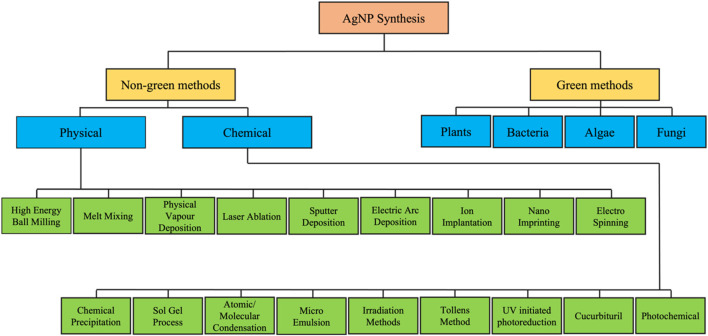
Illustrative description of a number of distinct approaches to the manufacture of silver nanoparticles.

**FIGURE 2:**
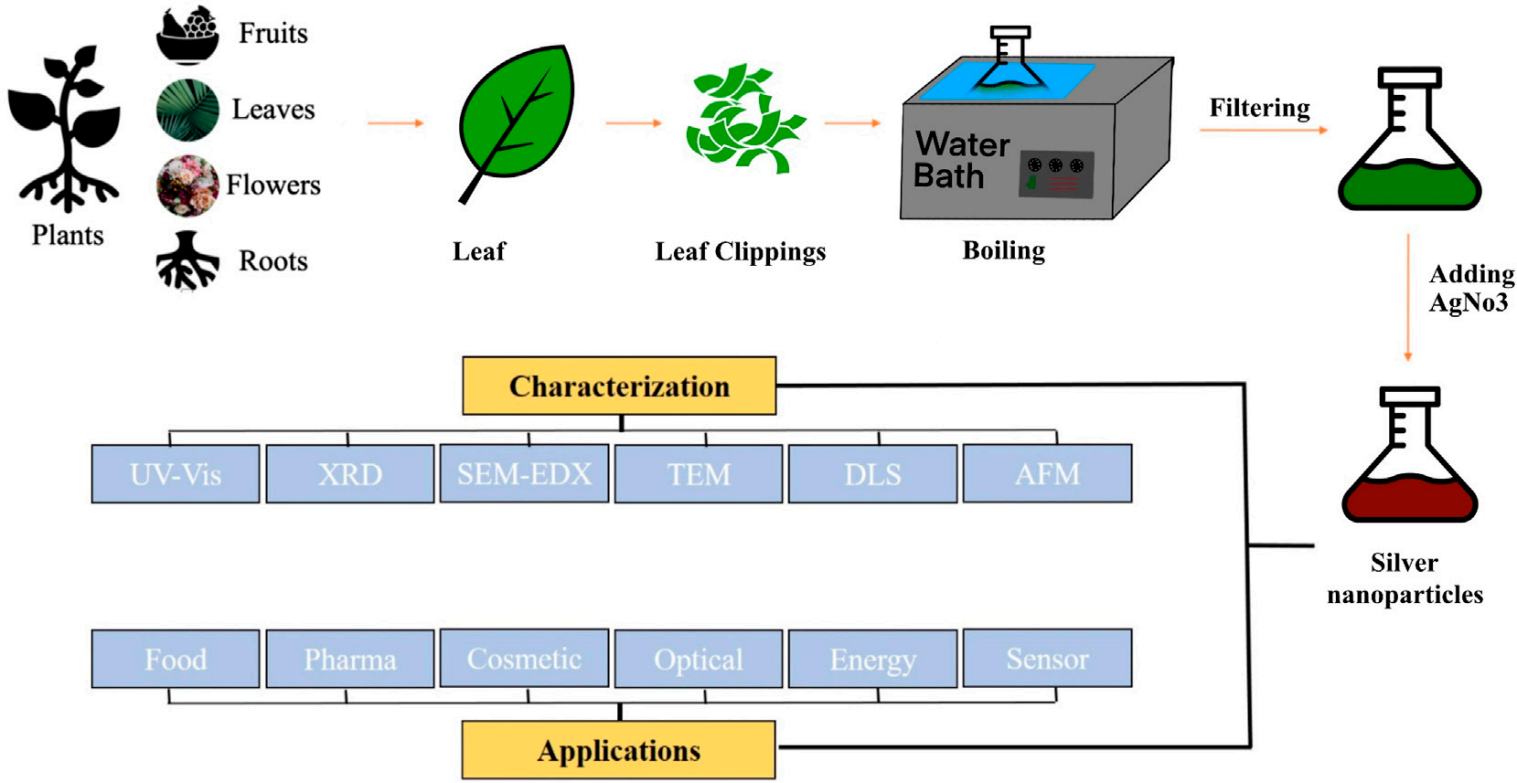
Steps involve in the biosynthesis of silver nanoparticles using plant extract, as well as the characterization of the nanoparticles and consideration of possible applications for them.

The production of AgNPs can be accomplished through a wide variety of approaches, including chemical, physical, and even biological techniques. Figure 1 presents a summary of the many different approaches that can be taken to accomplish the production of silver nanoparticles. Chemical techniques can be further subdivided into electrochemical, irradiation-assisted chemical, pyrolysis, and chemical reduction methods. To synthesize AgNPs in a solution, a metal precursor, reduction agents, and agents that can either cap or stabilize the product are required. Ascorbic acid, alcohol, borohydride, sodium citrate, and hydrazine compounds are examples of chemicals that are used frequently in reducing reactions (Iravani et al., 2014). Physical techniques, on the other hand, do not call for the use of any potentially lethal or highly reactive chemicals and are significantly faster compared to other approaches. Techniques that are considered to be examples of physical processes include arc discharge, physical vapor condensation, energy ball milling techniques, and direct current magnetron sputtering. In comparison to chemical methods, the physical methods require a greater amount of energy, but they produce nanoparticles with a more uniform size distribution (Naganthran et al., 2022). Therefore, the scientific community began to recognize biological methods for the synthesis of AgNPs from plant component extracts and/or microorganisms as a viable alternative to chemical and physical methods of production. Biological methods offer several benefits, including lower overall costs, less impact on the surrounding environment, greater ease of implementation, and the capacity to be easily ramped up to achieve higher outputs or yields. The biological synthesis of silver and silver oxide nanoparticles with the assistance of plant materials has become very widespread in the field of nanotechnology as a result of the superior properties that these nanoparticles possess. A rundown of the methods that can be implemented to accomplish the manufacturing of silver nanoparticles by making use of plant materials is provided in Figure 2.

Plants and their constituents contain a diverse range of compounds, including carbohydrates, lipids, pigments, alcoholic compounds, alkaloids, vitamins, polysaccharides, proteins, and various secondary metabolites. In the field of synthesizing nanoparticles from metal salts, these compounds serve versatile roles as reducing agents, capping agents, or stabilizing agents, producing minimal to no harmful by-products (Ijaz et al., 2022). Although the precise method and molecules involved in plant-mediated synthesis are not fully understood, there is a hypothesis that electrostatic interactions between silver ions and chemicals in plant extracts initiate bioreduction (Marslin et al., 2018).

A common mechanism involves the reduction of bulk silver by proteins in plant samples (Li et al., 2007). Plant extracts facilitate this reduction, forming bonds between biomolecules and the nanoparticle surface, thereby enhancing stability (Makarov et al., 2014). Capping agents play a vital role in preventing aggregation and coagulation by binding to the surface of silver nanoparticles. Various capping agents allow for precise control over AgNP size and shape. While stabilizing agents share similarities with capping agents, their primary function is to impede the oxidation of AgNPs. Given the high reactivity of silver, oxidation can result in the formation of undesirable silver oxides, compromising nanoparticle stability and properties.

Reducing agents are pivotal in converting silver ions (Ag+) to silver nanoparticles (Ag0) by contributing electrons to Ag+. In green synthesis, plant extracts serve as rich sources of these agents, actively participating in the processes of AgNP reduction, capping, and stabilization. Examples of phytochemicals involved in these processes are provided in Table 1.

**TABLE 1:** Examples of phytochemicals involved in plant-mediated synthesis of silver nanoparticles as capping, stabilizing, and reducing agents.

Plants	Capping, stabilizing, and reducing agents	References
*Acalypha indica*	Quercetin	Krishnaraj et al. (2010)
*Achyranthes aspera*	Polyols	Elumalai et al. (2016)
*Aegle marmelos*	Tannin	Rao and Paria (2013)
*Azadirachta indica*	Flavonoids, terpenoids	Ahmed et al. (2016a)
*Syzygium cumini*	Polyphenols	Kumar et al. (2010)
*Hibiscus rosa-sinensis*	Carboxylate ion groups	Nayak et al. (2015)
*Solanum xanthocarpum*	Alkaloids, phenolic, sugars	Sengottaiyan et al. (2016)
*Trianthema decandra*	Saponin	Geethalakshmi and Sarada (2013)

## 3 Characterization of silver nanoparticles

**FIGURE 3:**
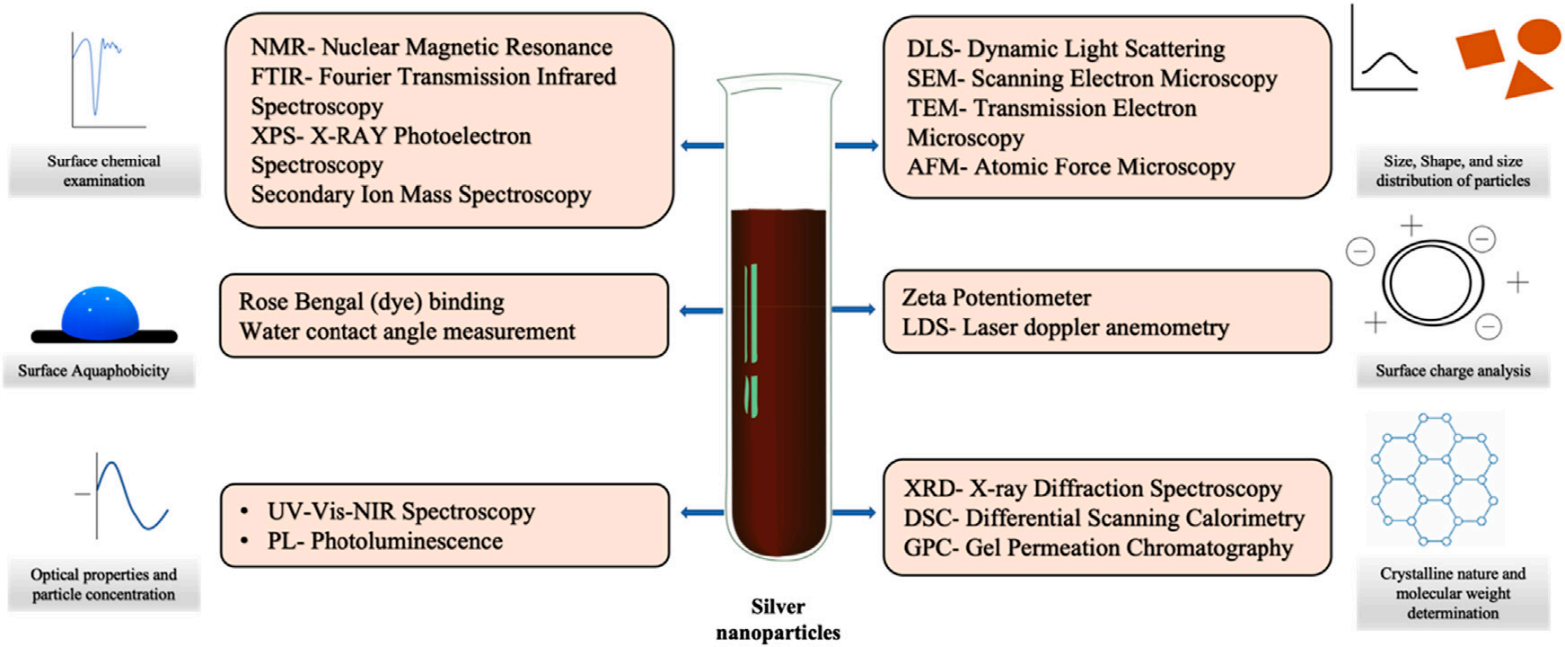
The characterisation of silver nanoparticles can be accomplished through the use of a wide range of distinct physiochemical techniques.

Characterization is a crucial phase that silver nanoparticles go through after they have been successfully generated (or produced). The importance of this procedure cannot be overstated when it comes to comprehending their functional qualities, morphology, surface chemistry, surface area, and other possible variations in their composition. As shown in Figure 3, the process of characterizing silver nanoparticles often includes the combination of several different microscopic and spectroscopic approaches.

A complete characterization of these nanoparticles was accomplished by the utilization of the following methods:

Ultraviolet-visible spectroscopy, often known as UV-Vis spectroscopy, is a technique that is utilized to evaluate the properties of metallic nanoparticle products. The ability of the nanoparticles to maintain their stability and the rate at which they are formed are both monitored in order to do this. In addition, UV-Vis spectroscopy is utilized in order to collect information concerning the phenomena that are referred to as surface plasmon resonance (SPR) in semiconductor nanoparticles. In particular, silver nanoparticles (AgNPs) exhibit a peak in UV–visible absorption within the range of 400–500 nm as a result of surface plasmon resonance (SPR). Consequently, the investigation of this feature contributes to the identification of silver nanoparticles (Chandraker et al., 2021).

In order to determine the molecular structure as well as the crystalline arrangement of metallic nanoparticles, X-ray diffraction analysis is a technique that involves directing X-rays deep into a substance (Chandraker et al., 2021). It has been shown that silver nanoparticles exhibit clear and well-defined peaks at particular 2 theta angles, specifically at planes 122, 111, 200, 220, and 311. This is an important observation. It is because of this distinguishing characteristic that AgNPs are easier to identify (Jemal et al., 2017).

In the process of investigating and analyzing the surface chemistry of metal nanoparticles that have been manufactured, Fourier transform infrared spectrophotometry (FTIR) is an extremely important technique. Specifically, it makes it possible to investigate the role that biomolecules play in the complex process of nanoparticle production. The Fourier transform infrared spectroscopy (FTIR) is an indispensable tool for investigating the surface chemistry of these nanoparticles as well as the contributions of biomolecules throughout the bio-fabrication process. According to Chandrakar et al. (2021), this highly developed method is quite helpful in gaining an understanding of the chemical interactions and compounds that occur on the surfaces of nanoparticles.

EDX, which stands for energy-dispersive X-ray spectroscopy, is a valuable technology that is utilized to determine the elemental composition of the material. In order to ascertain the elemental composition of any nanoparticle, it is possible to make use of the distinct peaks in the X-ray spectra. These peaks are generated by the different atomic structures that are associated with each element. This technique is an essential instrument for accurately detecting the elemental composition of the substance that is being investigated, which provides insights on the chemical makeup of the substance (Barabadi et al., 2021).

Scanning electron microscopy, often known as SEM, is a sophisticated imaging technology that is largely utilized for the purpose of determining the structure, size, and surface features of nanoparticle applications. A dual technique that makes use of scanning electron microscopy and energy-dispersive X-ray spectroscopy (EDX) is utilized in order to conduct a comprehensive examination of the chemical composition as well as the morphology of the silver particles. This combination method is quite helpful in acquiring significant insights into the chemical makeup of the silver particles as well as the physical characteristics of the particles themselves.

Transmission electron microscopy (TEM) serves as an analytical technique that provides intricate details concerning the crystallographic characteristics, morphology, and dimensions of nanoparticles. This method is crucial for offering highly precise insights into both the structural and physical attributes of nanoparticles, encompassing their crystallography, size, and shape (Chandraker et al., 2021).

Dynamic light scattering (DLS) is an analytical approach that not only facilitates the assessment of size distribution among molecules but also allows for the determination of the average size of nanoparticles while they are suspended in a liquid medium. Furthermore, this method proves to be highly effective for scrutinizing the size distribution among molecules (Barabadi et al., 2021).

The zeta potential analyzer plays a pivotal role in assessing the surface charge attributes of nanoparticles, making it an essential tool for determining their surface charge potential. This instrument is instrumental in evaluating the electrokinetic properties of nanoparticles, providing valuable insights into their surface characteristics and behavior (Chandraker et al., 2021).

X-ray photoelectron spectroscopy (XPS) stands among various spectroscopic techniques utilized to investigate the elemental composition of a sample, exposing it to light irradiation through proton energy. This technique proves to be a potent tool for delving into the surface characteristics of nanoparticles. Its application extends to discerning both the elemental composition and the chemical state of elements on the nanoparticle surface. This valuable information aids in evaluating the uniformity of nanoparticles and gaining insights into their surface chemistry. In a study conducted by Olivieri and Brown (2016), the investigation of a colloidal nanoparticle with a core-shell structure in an aqueous solution was undertaken using XPS. This particular nanoparticle featured a gold core surrounded by a silver shell. The XPS data uncovered that the gold core was enveloped by a layer of silver atoms, maintaining their metallic state. This research showcases the capacity of XPS to enable atomic-level examinations of nanoparticle structure.

Brunauer–Emmett–Teller (BET) theory or BET adsorption equation is employed to calculate the specific surface area of AgNP’s and gives us important information regarding physical structure which in turn helps to understand how nanoparticles will interact with the environment. The surface area of a substance is commonly connected with many attributes, including moisture retention, catalytic activity, dissolution rates, and shelf life. One of the most popular techniques for characterizing materials is surface area analysis, which is essential to the design and production of solids (Sinha et al., 2019).

These diverse characterization methods collectively offer a comprehensive and in-depth understanding of the properties and attributes of silver nanoparticles. This knowledge is pivotal for harnessing their potential across various applications and fields.

## 4 Medicinal properties of silver nanoparticles

In recent years, extensive research and a mounting body of evidence have shed light on the multifaceted medicinal properties of silver nanoparticles, positioning them as promising assets in the realm of medicine and pharmaceuticals. These properties span a diverse spectrum of therapeutic characteristics, encompassing a wide range of applications. Notably, silver nanoparticles have demonstrated their effectiveness in combating bacterial, fungal, and viral infections, inhibiting biofilm formation, regulating diabetes, exhibiting anti-cancer potential, combating parasitic infections, acting as antioxidants, and alleviating inflammatory conditions. The versatility of these medicinal attributes is further underscored by their successful synthesis through the use of various medicinal plants, adding to the allure of silver nanoparticles in medical and pharmaceutical contexts.

### 4.1 Antimicrobial property

Anti-microbial substances are substances that can kill microorganisms such as bacteria, and fungi, or stop them from growing and causing disease. According to WHO (World Health Organisation) Antimicrobial-including antibiotics, antifungal, antiparasitic, and antiviral are the medicines that are used to prevent and treat infections in humans, animals, and plants. Penicillin, the serendipity of 1928 is credited to Alexander Flemming whose discovery had a huge impact on antibacterial treatment since World War 1 (1948) till today. This discovery led to the wide use of antibiotics to treat different infections worldwide, however, soon after, microbes started to develop resistance against penicillin, and similar observations were recorded regarding other antibiotics as well. The prevalence of drug resistance among microorganisms made it a global issue to find alternatives to antibiotics and silver nanoparticles were among the mix which had the potential to fight against these microorganisms. Silver nanoparticles have been widely used for antimicrobial activity for many years because of their wide applications against a plethora of microorganisms including bacteria, fungi, and viruses. In recent studies and experiments, the observed antibacterial activity has been determined with the help of methods such as disc diffusion, well diffusion, Minimum Inhibitory Concentration (MIC), and Minimum Bactericidal Concentration (MBC). In a study, Almatroudi et al. (2020) reported that silver nanoparticles synthesized using seed extract of *Nigella sativa* were of size 8–80 nm and showed bacteriostatic and bactericidal activity against a variety of bacteria including *Enterococcus faecalis*, *Eshcherichia coli*, *Klebsiella pneumoniae*, *S. aureus*, and *P. aeruginosa*. In another study by Rao and Savithramma, (2011) the silver nanoparticles were synthesized from the leaf extract of *Svensonia hyderobadensis*, and stem bark extracts of *Boswellia ovalifoliolata* and *Shorea tumbuggaia* where AgNPs synthesized from leaf extract of *Streptomyces hyderobadensis* inhibited the growth of *Pseudomonas* sp. and *Rhizopus* sp. while the AgNPs synthesized from bark extracts of *B. ovalifoliolata* and *S. tumbuggaia* showed toxicity towards *Klebsiella* and *Aspergillus* sp., *Pseudomonas* sp. and *Fusarium* sp. respectively.

**FIGURE 4:**
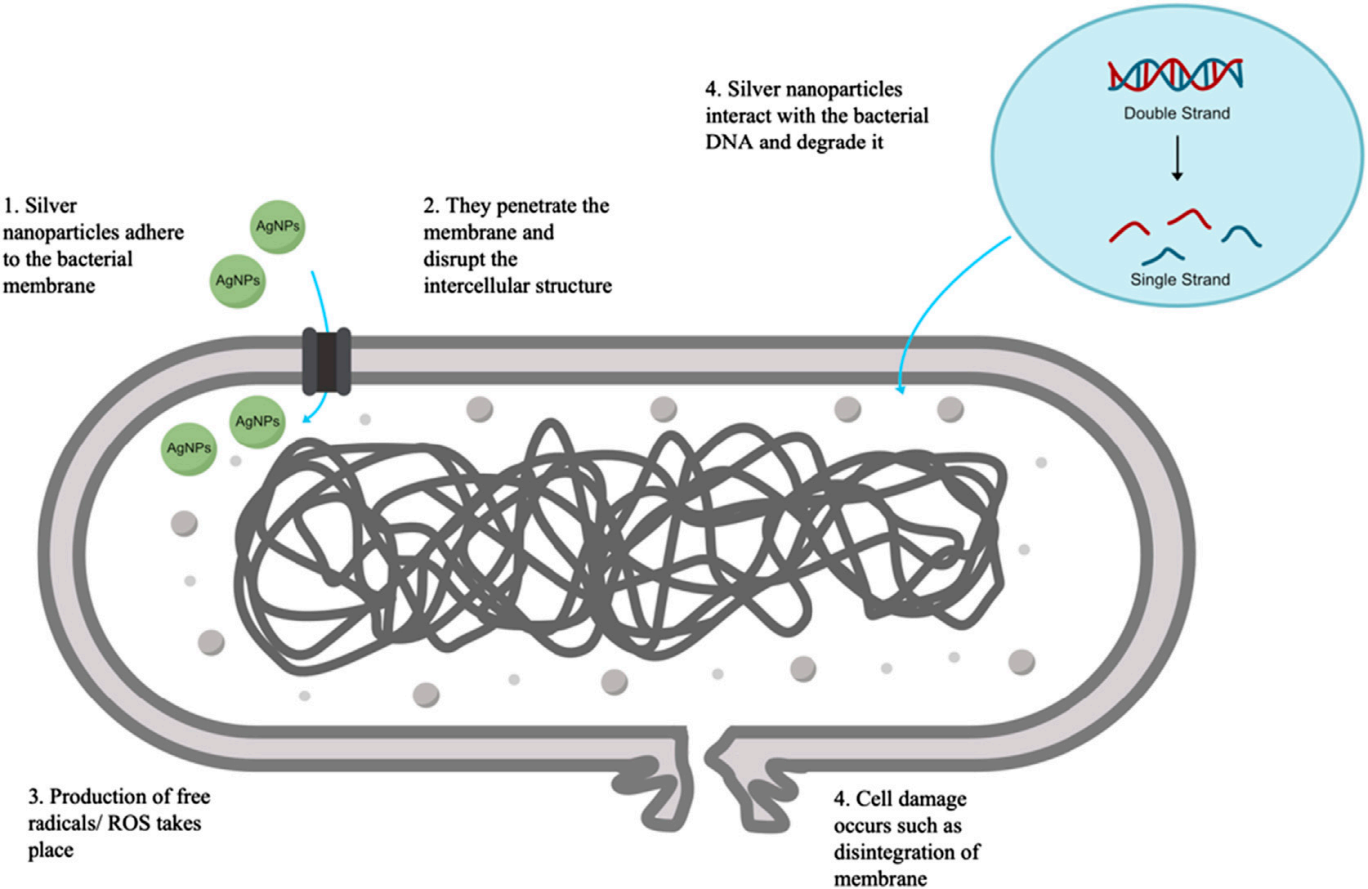
Illustration depicting the likely mode of action of silver nanoparticles as antibacterial agents, including the rupture of membranes, creation of free radicals, and damage of DNA, all of which lead to the death of cells.

Although it is not known how the antibacterial properties of silver nanoparticles actually work, reactive oxygen species generated from AgNPs are considered the primary agent that contributes to the disruption of cell membranes and the modification of DNA that results from these disruptions. The interaction of sulfur and phosphorus found in DNA with silver ions has the potential to impede the replication of DNA as well as the reproduction of cells. It is possible for silver ions that have been released by nanoparticles to either bind to the cell wall due to electrostatic forces, which would make the cell wall more permeable, or they could pass through the cell wall or even cytoplasmic membranes and possibly accumulate in the pits of the cell wall, which would result in membrane denaturation (Duran et al., 2016; Khorrami et al., 2018). Because of their nanoscale size, these silver nanoparticles have the ability to pass through the cell walls of bacteria and alter the structural integrity of the cell membrane. Gram-negative bacteria, which have a cell wall that is significantly thinner than Gram-positive bacteria, are more vulnerable to the effects of silver nanoparticles (Meikle et al., 2020). In addition, silver ions have the ability to denature ribosomes and impede the production of proteins (Liao et al., 2019). There is also the possibility that reactive oxygen species (ROS) produced by broken electron transport chains can cause membrane disruption in mitochondria, thereby bringing an end to the production of ATP. Additionally, the inactivation of respiratory enzymes on the cytoplasmic membrane caused by silver ions is another potential cause for the cessation of ATP production (Ramkumar et al., 2017; Liao et al., 2019; Yin et al., 2020). A diagrammatic representation of the hypothesized mechanism by which AgNPs exert their antibacterial effects is provided in Figure 4.

Along with antibacterial properties, it is equally important to find novel agents for fungal infections as well. Fungi are the cause of numerous pathogenic diseases in both plants and animals, such as ergot of rye and aspergillosis, respectively, and it is important to find novel agents for treating both types of infections. The disease is caused by fungi when they inadvertently breach the membrane that protects the host and then develop a virulence mechanism that allows them to multiply within the host cell. Due to the severity of these conditions, it is imperative that alternative treatments to chemical and synthetic medications be sought. In a recent study silver nanoparticles were manufactured using a bacterial strain of *Bacillus* sp. MB353 (PRJNA357966), The nanoparticles ranged in size from 49 to 53 nm and exhibited remarkable antifungal activity against *Aspergillus niger, Aspergillus fumigatus,* and *Fusarium soleni* (Khan et al., 2020). According to the findings of previous research, the primary reason that AgNPs have antifungal activity is that they inhibit the germination of conidiospores and the growth of mycelial networks. According to the findings of research conducted by Kim et al. (2008), the antifungal activity may be caused by a disruption of the cell membrane as well as an inhibition of the normal budding process. Several other examples of plant-mediated synthesized silver nanoparticles with antibacterial and antifungal activity are mentioned in Table 2.

**TABLE 2:** List of AgNPs with antibacterial and antifungal activity synthesized using plant extracts.

Plant name	Shape and size of AgNP	Antimicrobial property against	References
*Nyctanthes* *arbortristis*	Spherical and oval, 5–20 nm	Escherichia *coli*	Gogoi et al. (2015)
*Carica papaya*	Spherical, 5 and 40 nm	*Staphylococcus aureus, Bacillus subtilis*	Banala et al. (2015)
*Mangifera indica*	Quasi Spherical, 30.51 ± 5.3	*Pseudomonas aeruginosa*	Hai et al. (2022)
*Fragaria ananassa*	Spherical, 7–65 nm	*Pseudomonas aeruginosa* and *Bacillus licheniformis*	Umoren et al. (2017)
*Cestrum nocturnum*	Spherical, 20 nm	*Vibrio cholera* and *Enterrococcus* *faecalis*	Keshari et al. (2020)
*Brassica oleracea*	Spherical, 20 nm	*Streptococcus pneumonia ATCC 10015, Staphylococcus aureus ATCC 6538*	Ansar et al. (2020)
*Helicteres isora*	Spherical, 16–95 nm	*Salmonella typhi* and *Pseudomonas aeruginosa*	Bhakya et al. (2015)
*Piper longum*	Spherical, 46 nm	*Bacillus cereus* and *Staphylococcus aureus*	Reddy et al. (2014)
*Bergenia ciliata*	Spherical, 35 nm	*Aspergillus niger, Aspergillus fumigatus*	Phull et al. (2016)
*Amaranthus retroflexus*	Spherical, 10–32 nm	*Fusarium oxysporum, Alternaria alternata, Macrophomina phaseolina*	Bahrami-Teimoori et al. (2017)
*Brassica rapa L*	Spherical, 39.5 nm	*Gloeophyllum abietinum, Gloeophyllum trabeum*	Narayanan and Park (2014)
*Svensonia hyderabadensis*	Spherical, 45 nm	*Aspergillus niger*	Rao and Savithramma (2011)
*Malva parviflora*	Spherical, 50 nm	*Helminthosporium rostratum, Fusarium solani, Fusarium oxysporum* and *Alternaria alternate*	Al-Otibi et al. (2021)

### 4.2 Anti-biofilm activity

A community of microorganisms that have attached themselves to a surface and are shielded by a matrix of extracellular polymeric compounds is referred to as a “biofilm” These biofilms constitute a serious risk in a number of different industries, including the healthcare industry, the petroleum industry, the shipping industry, and others, where they cause significant economic loss in addition to other issues. In recent years, it has come to light that nanoparticles can demonstrate potent anti-biofilm characteristics by specifically targeting several stages of the production of biofilm (Martinez-Gutierrez et al., 2013). It has been extensively noted that, in comparison to other nanoparticles, AgNPs have very potent anti-biofilm effects against a wide variety of bacteria. In one of the studies, it was found that silver nanoparticles had potent anti-biofilm action against bacterial species such as *P. aeruginosa, Escherichia coli*, and *S. aureus* (Mohanta et al., 2020). Another study, conducted by Almatroudi et al. (2020), found that the formation of biofilm was inhibited by silver nanoparticles (Synthesized using seed extract of *N. sativa*) by 88.42% for *E. faecalis*, 84.92% for *E. coli*, 81.86% for *K. pneumoniae*, 82.84% for *S. aureus*, and 49.9% for *P. aeruginosa*. AgNPs treatment during the formation of biofilm has been reported in some studies to inhibit the adherence, colonization, and formation of microbial biofilm on different surfaces. In the current study, violacein production in *Chromobacterium violaceum* assessed by the agar well diffusion method was inhibited by silver nanoparticles (AgNPs) synthesised from the peels of oranges, gingers, lemons, cinnamon, corn silk and pomegranates. This demonstrated the AgNPs’ anti-QS characteristics (Sheikh and Tale, 2017). However, the antibiofilm property can vary greatly depending on the method of synthesis, stability, and size of silver nanoparticles (Martinez-Gutierrez et al., 2013). Table 3 provides a summary of a few examples of the anti-biofilm activity of plant-mediated synthesized silver nanoparticles.

**TABLE 3:** Antibiofilm activity of silver nanoparticles synthesized using plants.

Plant name	Shape and size of AgNP (nm)	Anti-biofilm properties against	References
*Glochidion lanceolarium*	Spherical, 92.3	*Pseudomonas aeruginosa, Escherichia coli,* and *Staphylococcus aureus*	Mohanta et al. (2020)
*Semecarpus anacardium*	Spherical, 62.72	*Pseudomonas aeruginosa, Escherichia coli,* and *Staphylococcus aureus*	Mohanta et al. (2020)
*Bridelia retusa*	Spherical, 74.56	*Pseudomonas aeruginosa, Escherichia coli,* and *Staphylococcus aureus*	Mohanta et al. (2020)
*Anethum graveolens*	Spherical, 9.67	*Staphylococcus aureus, Enterococcus faecalis Escherichia coli, Klebsiella pneumoniae, Pseudomonas aeruginosa*	Rolim et al. (2019)
*Camellia sinensis*	Spherical, 11.3	*Staphylococcus aureus, Enterococcus faecalis Escherichia coli, Klebsiella pneumoniae, Pseudomonas aeruginosa*	Rolim et al. (2019)
*Azadirachta indica*	Spherial, 12.27–27.20	*Candida tropicalis*	Al-Aboody (2019)
*Dononaea* *viscosa*	Spherical, 40–55	*Candida* sp.	Muthamil et al. (2018)
*Hyptis* *suoveolens*	Spherical, 40–55	*Candida* sp.	Muthamil et al. (2018)

The management of biofilms is crucial in combating bacterial infections, and various strategies have been explored to achieve this goal. One approach is the eradication of bacterial cells, either within biofilms or in the planktonic stage. Another strategy involves disrupting quorum sensing, a key communication mechanism among bacteria, or preventing the formation of biofilms altogether (Sen et al., 2021).

Silver nanoparticles (AgNPs) have emerged as a promising tool for treating biofilm-prone surfaces and preventing biofilm development (Besinis et al., 2014). The unique properties of AgNPs allow them to act synergistically with conventional antibiotics. Existing literature supports the idea that this synergy is attributed to AgNPs disrupting microbial cell membranes and causing DNA damage. This dual mechanism enhances the effectiveness of antibiotic treatments and holds the potential to overcome antibiotic resistance, a growing concern in microbial infections (Swidan et al., 2022). The combined action of AgNPs and antibiotics presents a promising avenue for improving treatment outcomes and addressing the challenges posed by antibiotic-resistant strains.

### 4.3 Cytotoxic activity

Cancer, according to the National Cancer Institute in the United States, is a disease in which body cells begin to grow uncontrollably and may spread to other parts of the body. Cancer is the leading cause of death worldwide, accounting for nearly 10 million deaths in 2020 (Ferlay et al., 2020). Among various cancers, breast cancer accounted for the newest cases among all cancers, with 2.26 million new cases, followed by lung cancer with 2.21 million new cases and lung cancer contributing to 1.80 million deaths. Among all cancer-associated deaths, 22% of cancer deaths are attributed to tobacco use, 10% to excessive drinking of alcohol, poor diet, and lack of physical activity, 15% to bacterial and viral infections such as Hepatitis B and C, human papillomavirus infection, Epstein-Barr virus, and HIV, and 5%–10% to inherent genetic disorders (ACS, 2018). Other things like ultraviolet and ionizing radiation, chemicals like asbestos and arsenic can all contribute to the development of cancer in humans.

The treatment of cancer typically consists of surgery, radiation therapy, chemotherapy, hormonal treatment, targeted biological therapies, or a combination of these; however, these treatments come with their own set of side effects that can range from weight loss and nausea to the loss of all hair and, in the most severe cases, cardiotoxicity and even death. The cytotoxic capability of AgNPs against cancerous cells has been extensively reported in recent years, and as a result, these nanoparticles can be utilized in the treatment of cancer. *Elaeodendron croceum* stem bark extract was used in one of the studies to synthesize AgNPs, which later demonstrated *in vitro* cytotoxic activity against MDA-MB-231 breast cancer cell lines with an IC50 value of 138.8 3.98 g/mL (Odeyemi et al., 2020). In yet another piece of research, the substance of the leaf of *Brachychiton populneus* was utilized in the production of AgNPs. The particles that were produced had a cubical structure and a diameter of 12 nm, and they demonstrated potent cytotoxic activity against HEK 293 (Human embryonic kidney 293 cells) and U87 (Uppsala 87 Malignant Glioma) cell lines with an IC50 value of 64.85 g/mL and 168.97 g/mL, respectively (Naveed et al., 2022). Sre et al. (2015) conducted research on AgNPs that were produced using an aqueous root extract of *Erythrina indica lam*. They made spherical nanoparticles that ranged in size from 20 to 228 nm and had a spherical shape. During the course of this research, biosynthesized AgNPs were put through an MTT test where they demonstrated high cytotoxicity, as they were able to kill more than 70 percent of the MCF-7 cells and 80 percent of the HEPG2 cells, respectively. In a separate piece of research, silver nanoparticles (AgNPs) were produced using the extract from the *Conocarpus Lancifolius* plant by employing a bottom-up approach. These AgNPs were found to have a spherical shape and ranged in size from 5 to 30 nm. The apoptotic potential of AgNPs was investigated in this study using the MTT assay on MDA-MB-231 cell lines, which revealed a death rate of greater than 80% in these cells. In addition, the biocompatibility of these AgNPs was investigated by performing RBC lysis on human erythrocytes. The results suggested that these AgNPs were stable and did not cause any toxicity (Oves et al., 2022). Additional plant-based AgNPs with cytotoxic activity are listed in Table 4.

**TABLE 4:** List of examples of plants based AgNPs having cytotoxic activity.

Plant used for synthesis	Shape and size of AgNPs	Cytotoxic activity	References
*Juglans regia*	Spherical, 31.4 nm	Breast cancer cell line (MCF-7)	Khorrami et al. (2018)
*Piper longum*	Spherical, 15–40 nm	Breast cancer cell line (MCF-7)	Jayapriya, et al. (2019)
*Phoenix dactylifera*	Spherical, 67 nm	Colon cancer cell line (LoVo)	Mohammed et al. (2018)
*Ferula asafoetida*	Spherical, 105.7 nm	Colon cancer cell line (LoVo)	Mohammed et al. (2018)
*Acacia nilotica*	Spherical, 100.4 nm	Colon cancer cell line (LoVo)	Mohammed et al. (2018)
*Calligonum comosum*	Spherical, 90.8 nm	Hepatoma G2 (HepG2), Colon cancer cell line (LoVo), and Breast cancer cell line (MDA-MB231)	Algebaly et al. (2020)
*Azadirachta indica*	Spherical, 34 nm	HepG2, LoVo, and MDA-MB231	Algebaly et al. (2020)
*Anthemis atropatana*	Spherical, 38.89 nm	Human colon cancer cell line (Ht29)	Dehghanizade et al. (2018)
*Tinospora cordifolia*	Spherical, 25–50 nm	Human lung adenocarcinoma cell line (A549)	Mittal et al. (2020)
*Cyperus conglomeratus*	Spherical, 70–100 nm	Breast cancer cell line (MCF-7)	Al-Nuairi et al. (2020)

**FIGURE 5:**
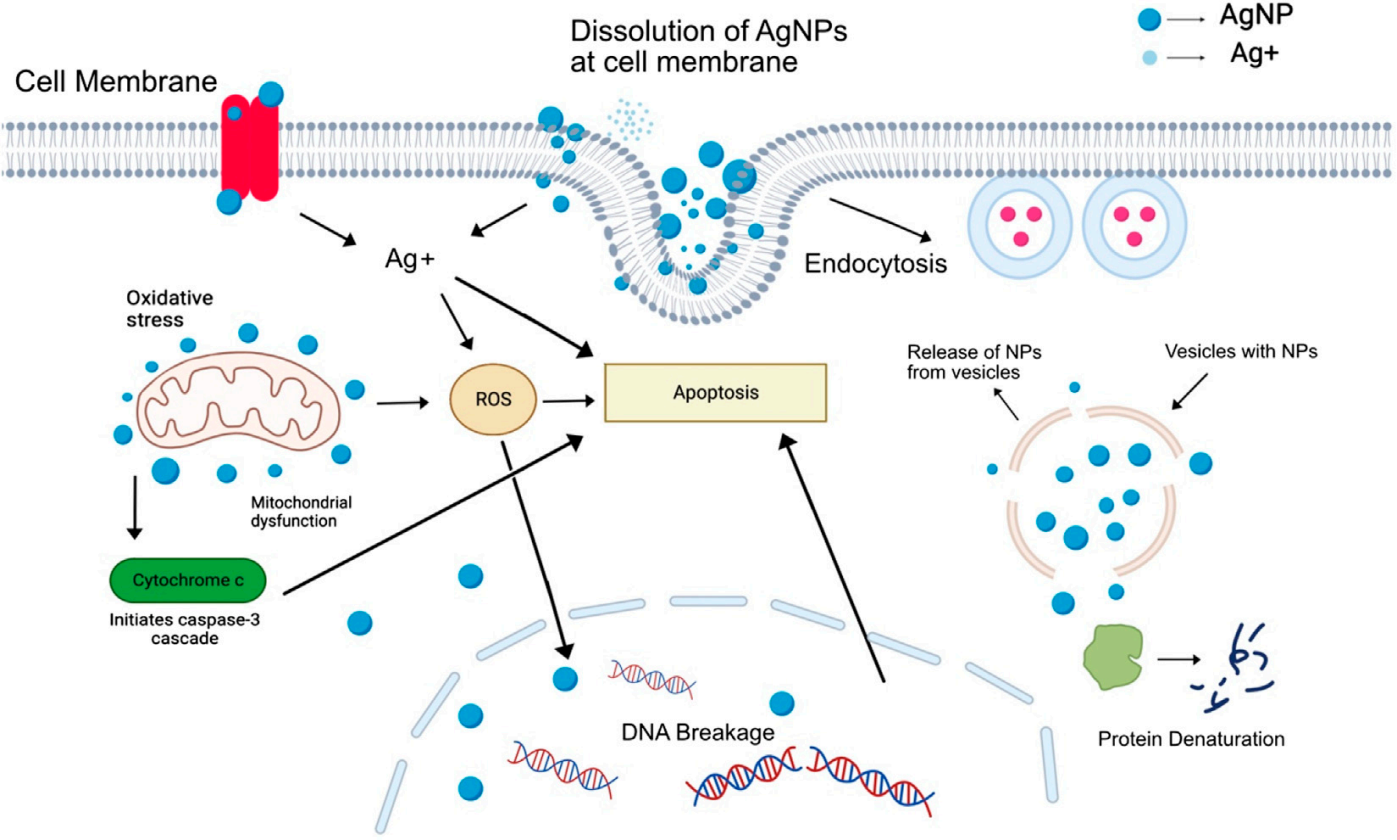
Illustration of the mechanism through which silver nanoparticles exert their anti-cancer effects by inducing oxidative stress and releasing silver ions.

Previous research has shown that nanoparticles have a greater ability to suppress cancer cells than bulk materials do. In addition, nanoparticles produced using conventional chemical and physical methods are much more dangerous than those produced using medicinal plants. These green silver nanoparticles enter the cell, where they inhibit essential cellular processes by enveloping the cell membrane and passing through it. Due to the fact that AgNPs are able to pass through the cell membrane of cancer cells, they are able to cause disturbances at the cellular, subcellular, and biomolecular levels (Gupta and Xie, 2018). It is presently believed that reactive oxygen species (ROS) are responsible for producing the cytotoxicity of AgNPs. This, in turn, is thought to result in decreased levels of the natural antioxidant glutathione, which in turn leads to an excessive amount of oxidative stress. Kim and Ryu (2013) reported that the treatment of cancerous MCF-7 and MCT cell lines with biologically synthesized silver nanoparticles led to substantial cytotoxicity. It was also discovered that the treatment led to oxidative stress, genotoxicity, and apoptosis in the cells. The release of reactive oxygen species (ROS) from silver nanoparticles has been shown in a number of other studies to be capable of causing DNA damage in cancerous cells, which can then eventually lead to apoptosis of those cells (Jain et al., 2021). In a different research endeavor, it was discovered that cancer cells that had been treated with AgNPs exhibited increased production of reactive oxygen species, in addition to depolarization of mitochondria, apoptotic cell population (sub-G1), and DNA disintegration (Kumari et al., 2020). Also, it has been shown that AgNPs can induce apoptosis in tumor cells by influencing the activity of signaling proteins, such as the caspase-3 cascade. The discharge of cytochrome c from mitochondria as a response to oxidative stress is the event that sets off this cascade (Xu et al., 2020). As was covered in the segment before this one, Figure 5 presents a visual representation of the various anticancer mechanisms of action that silver nanoparticles possess.

### 4.4 Wound healing

In the event of an injury, an organism will begin the process of wound healing in order to restore any damaged tissues. It is necessary for the repair and upkeep of the structural and functional integrity of the tissues, making it an important component overall. The process of wound recovery can be broken down into three distinct stages: the inflammatory phase, the proliferative phase, and the remodeling phase (Gonzalez et al., 2016). In addition to the nature, location, and degree of severity of the wound, as well as the patient’s general health and medical history, the method of wound healing that is employed is determined by these factors. These approaches to wound healing are classified as either primary (using staples, stitches, or adhesive material), secondary (allowing the wound to remain open to permit healing from the bottom up), or tertiary (the wound is left open initially, and then closed after the removal of contaminated tissue) (Chhabra et al., 2017). Methods such as skin substitutes, grafts, negative pressure wound treatment, and medications are also used as necessary in the process of wound healing. These methods are utilized depending on the severity of the wound (Hopf et al., 2001).

In the course of medical history, silver has been applied to wounds in a variety of different ways, such as when it was included in a concoction of dry granules that consisted of a variety of metals and applied to wounds after they had been cleaned. In the early 20th century, medical practitioners frequently turned to the use of colloidal silver, which was made up of nothing but purified silver and water. During World War II, silver foil dressings were used as an antiseptic additive in the treatment of infected wounds. These dressings were used to treat infected wounds. The application of silver chloride in an aqueous solution as a topical treatment, along with dressings made of silver nitrate (Spear, 2010). Silver is an effective antimicrobial agent that can eliminate infections caused by a broad variety of bacteria, fungi, and viruses. In one of the studies, wound healing properties of silver nanoparticles were compared with wounds treated with silver sulfadiazine or wounds that were left untreated. The researchers discovered that wounds treated with silver nanoparticles mended more quickly than wounds treated with silver sulfadiazine or wounds that were left untreated. The research was conducted by measuring the wound bed throughout the course of the healing process. It took an average of 28 days for wounds that were left untreated to recover, but wounds that were treated with silver sulfadiazine healed in an average of 24 days, and wounds that were treated with silver nanoparticles healed in an average of 18 days. It was discovered that AgNPs increase the proliferation of keratinocytes, which are responsible for the re-epithelialization process that occurs during wound recovery (Liu et al., 2010).

In today’s contemporary times, nanocomposite topical ointments that are biocompatible in their natural state have also been developed for the treatment of wounds and scars such as various composite silver nanoparticle-based ointments, namely, NanoSALV and Silvex Wound Gel which contain AgNPs derived from plants making them even more sustainable. These gel ointments are marketed as an effective aid in wound healing, infection control, and pain relief. (Gobush, 2022; Ho, 2023). Because they have a reduced risk of toxicity, silver nanoparticles are quickly replacing the use of silver sulfadiazine in scientific research (Mohseni et al., 2019). In some instances, excessive use of medications that contain silver sulfadiazine has been reported to cause blistering of the skin, fever, peeling of the skin, and a skin condition known as argyria, which is characterized by a discoloration of the skin. (Fuller, 2009). In other studies, dressings made with AgNPs have been put through rigorous testing to determine whether or not they are capable of delivering broad-spectrum antibacterial protection in a timely and efficient fashion (Wilkinson et al., 2011), a wound dressing with the commercial name of Acticoat Silver Antimicrobial Barrier Dressing by Smith and Nephew uses silver nanotechnology to create an optimal wound environment for faster healing and minimal infection using a nanocrystalline coating of pure silver which produces a silver-coated polyethylene film that releases silver constantly over a period of several days (Silver et al., 2007). The anti-inflammatory potential of silver nanoparticles can also be easily utilized through the process of covering cotton dressing with silver nanoparticles and incorporating them into the dressing. Additionally, because the strong antimicrobial potential of silver and microbial resilience to silver is so low, using silver coating in dressings brings the risk of infection down to a significant degree (Ahn et al., 2019). Table 5 provides additional instances of plant-based SNPs that have wound-healing properties and mentions these examples.

**TABLE 5:** List of examples of plant based AgNPs with wound healing properties.

Plant	Shape and size of AgNPs (nm)	Form used	Studied using	References
*Curcuma longa*	Spherical, 15–40	AgNP loaded cell lines	*In vitro* wound scratch assay using L929 fibroblast cell lines	Maghimaa and Alharbi (2020)
*Catharanthus roseus*	Spherical, 80–250	Nano-formulation	Mice excision wound model	Lakkim et al. (2020)
*Azadirachta indica*	Spherical, 40–80	Nano-formulation	*In vivo* mice excision wound model	Lakkim et al. (2020)
*Scutellaria barbata*	Spherical, 20–40	Coated cotton wound dressing	*In vitro* wound scratch assay	Veeraraghavan et al. (2021)
*Orchidantha chinensis*	Spherical, −25	AgNP coated dressing	*In vivo* mice excision wound model	Wen et al. (2016)
*Mimosa pudica*	Uneven Morphology, 〜 9.3–10.6	AgNP hydrogel	*In vivo* mice excision wound model	Muhammad et al. (2017)
*Stachys lavandulifolia*	Spherical, 20–40	AgNP ointment	*In vivo* mice excision wound model	Zangeneh et al. (2019)
*Arnebia Nobilis*	Spherical, 40–70	AgNP hydrogel	*In vivo* mice excision wound model	Garg et al. (2014)
*Boletus edulis*	87.77	Different concentrations of AgNP	*In vitro* wound scratch assay using L929 fibroblast cell lines	Kaplan et al. (2021)
*Pongamia pinnata*	Spherical, 20–60	AgNP loaded gel	*In vivo* mice excision wound model	Paul and Londhe (2019)

### 4.5 Bone healing

Millions of people suffer from bone-related conditions every year, including orthopedic infections, degenerative and hereditary diseases, malignant conditions, and fractures. These bone-related conditions can be quite diverse and complicated. The bones of the skeleton provide mechanical support for the joints, tendons, and ligaments of the body. They also protect vital organs from damage and act as a reservoir for calcium and phosphate, which helps the body maintain a healthy mineral homeostasis (Ralston, 2013). In most cases, bone transplants are performed to replace or repair severe osseous tissue defects caused by conditions such as genetic deformities, cancer, or injuries that cannot be repaired (Zhang et al., 2017). Infections that are associated with orthopedic procedures or bone implants are frequently accompanied by intense inflammatory processes, infections, the loss of the implant, and bone-destructive phenomena (Aurore et al., 2018a). Some of the most common microorganisms that can result in a bone infection are *S. aureus, Enterobacter* sp., *Mycobacterium tuberculosis,* and *Streptococcus pyogenes* (Garazzino et al., 2005). Osteoclasts are the cells that are responsible for initiating the normal process of bone remodeling by degrading any pathogen-induced deformities in the bone. These osteoclasts, on the other hand, are unable to eliminate bacteria and may instead serve as reservoirs for bacterial pathogens. If a bacterial infection takes place in a bone defect, the bone may lose some of its capacity to self-repair. According to the information presented in the prior portion, AgNPs possess an inherent antibacterial activity with broad-spectrum antimicrobial properties. AgNPs have the ability to both prevent and manage bacterial infections in orthopedic implants and bone, as well as prevent bacterial infections in human osteoclasts (Aurore et al., 2018b). Because silver nanoparticles possess antimicrobial properties, they have the ability to inhibit or reduce the development of biofilm in bacteria, such as *S. aureus* (Lu et al., 2016). Porous titanium implants that have been biofunctionalized by having a coating of AgNPs applied to them have the ability to release silver ions, which prevents the development of biofilm on the surface of the implant. These implants, as indicated by van-Hengel et al. (2017), do not cause any form of toxicity when they release silver.

One research found that Hydroxyapatite (Hap) scaffolds that were doped with silver nanoparticles exhibited a unique antibacterial activity. This activity has the potential to aid in the prevention of bacterial infections that are associated with bone implants (Zhou et al., 2015). Another study came to the same conclusion as Zhou and colleagues’ findings, that stainless steel coated with silver nanoparticles (AgNPs) helps prevent infections that are caused by orthopedic implants. The structural characterization of silver nanoparticles combined with one-of-a-kind hydroxyapatite (Hap) has been investigated for its application in orthopedic implants, and the findings indicated that the particles are suitable for implantation (Bharti et al., 2016). According to research that was conducted by Felix and Muthu (2016), spherical and 88 nm AgNPs that were synthesized from *Ormocarpum cochinchinense* were used in the production of bioscaffolds in order to speed up the process of injured bone’s healing and improve its overall quality. In comparison to other nanoparticles, the differentiation process of MC3T3-1 pre-osteoblast cells and the subsequent mineralization of bone-like tissue appears to benefit from the naturally occurring properties of AgNPs (Qing et al., 2018).

### 4.6 Larvicidal property

Larvicides are a type of insecticide that works by destroying the immature stages of insects, including the pupae and larvae, before the insects are able to mature into adults. Numerous diseases, including Japanese encephalitis, yellow fever, and dengue, among others, do not have a particular drug that can be used to treat them; therefore, the most viable option is vector control; therefore, larvicides are an important measure for the control of diseases. There is a large variety of chemically produced larvicides available; however, excessive use of these larvicides can lead to a variety of issues, including negative effects on the environment and the development of pesticide and larvicide resistance in the targeted vectors. Because of these issues, there is a pressing need for an alternative that is both safe and effective (Salunkhe et al., 2011).

One of the many uses for silver nanoparticles is as a larvicidal agent, which is just one of many uses (Govindarajan et al., 2016). In one of the studies, larval samples of *Anopheles subpictus* were exposed to AgNPs synthesized from the aqueous extract of *Nelumbo nucifera* at different concentrations for a period of 24 h. The AgNPs formed were truncated triangles, spherical, decahedral morphologies, and triangles ranging from 25 to 80 nm with an average size of 45 nm. The AgNPs treatment exhibited dose-dependent larvicidal activity against the larvae of *A. subpictus* (Santhoshkumar et al., 2011). In another study, Unissa et a., 2018 reported the larvicidal activity of silver nanoparticles synthesized from flower extracts of *Hibiscus vitifolius* against *Aedes aegypti*. According to the findings of another study, AgNPs that were manufactured using *Leucas aspera* (Thumbai), a plant that has been used for centuries due to the insecticidal and antipyretic properties it possesses, showed promising activity against the larva of the mosquito *A. aegypti*, which is responsible for the spread of the dengue virus. The produced AgNPs, which were primarily aggregated and had irregular, clustered forms, were evidently between 25 and 80 nm in size (Suganya et al., 2014). In urban India, *Anopheles stephensi* is the major vector for the spread of malaria, and AgNPs produced using the bark extractor of the tree *Holarrhena antidysenterica* have shown very strong larvicidal potential against larvae of *A. stephensi.* AgNPs varying from 40 to 60 nm in size and spherical, hexagonal, and triangular in shape were formed (Kumar et al., 2018). According to a study published by Gnanadesigan et al. (2011), AgNPs were synthesized using the aqueous extract of *Rhizophora mucronata* and evaluated for their larvicidal activity against *A. aegypti* and *Culex quinquefasciatus* mosquitoes. The authors found that AgNPs had a dose-dependent larvicidal effect on both species of mosquitoes, with the LC50 values being observed as 0.585 mg/L and 0.891 mg/L for *A. aegypti* and *C. quinquefasciatus* respectively. The average size of AgNPs formed was observed to be between 60–95 nm. Another study investigated the green synthesis of AgNPs using the aqueous extract of *Andrographis serpyllifolia* and their larvicidal activity against *C. quinquefasciatus* mosquitoes. The authors found that AgNPs had a dose-dependent larvicidal effect on *C. quinquefasciatus* mosquitoes, with an IC50 value of 68.889 μg/mL. With an average size of 24.1 nm and a range of 3.4 nm–71.6 nm, the silver nanoparticles were spherical in shape. The authors suggested that AgNPs could be a potential larvicidal agent for controlling mosquito-borne diseases and safeguarding human populations (Madhankumar et al., 2020). More examples of plant-based AgNPs having wound-healing properties are mentioned in Table 6.

**TABLE 6:** List of examples of plant based AgNPs with larvicidal properties.

Plant	Shape and size of AgNPs (nm)	Activity against	LC 50 value (ug/mL)	LC 90 value (ug/mL)	References
*Chomelia asiatica*	Triangular, 10	*Culex* *quinquefascuatus*	19.32	37.41	Muthukumaran et al. (2015)
*Solanum nigrum*	Spherical, 6.9	*Culex* *quinquefascuatus*	4.43	8.37	Adesuji et al. (2016)
*Annona muricata*	Spherical, 35	*Culex* *quinquefascuatus* *, Aedes aegypti, Anopheles stephensi*	18.77	35.72	Santhosh et al. (2015)
*Azadirachta indica*	Spherical, 17 ± 4	*Aedes aegypti*	1.25	-	Rasool et al. (2020)
*Citrullus colocynthis*	Spherical, 26 ± 5	*Aedes aegypti*	0.3	-	Rasool et al. (2020)
*Pergularia daemia*	Spherical, 44 to 255	*A. aegypti*	6.18 ± 0.38	12.95 ± 0.89	Patil et al. (2012)
		*A. stephensi*	5.91 ± 0.38	14.08 ± 1.08	
*Tinospora cordifolia* ** **Miers	55–80	*Pediculus humanus capitis*	12.46	-	Jayaseelan et al. (2011)
		*A. subpictus*	6.34		
		*C. quinquefasciatus*	6.96		

Although the mechanism that AgNPs use is not completely understood, a number of studies have led researchers to believe that it involves multiple modes of action. These modes of action include the following: In a research study, silver nanoparticles (AgNPs) were produced using the *Garcinia mangostana* plant, and then the larvicidal activity of these AgNPs against vectors of malaria and filaria was investigated. The authors speculated that morphological damage was caused by AgNPs because the particle size of the AgNPs allowed them to penetrate the cuticle and enter the individual cells of the larvae. This led them to hypothesize that AgNPs were responsible for the morphological damage. The researchers also noticed that AgNPs caused a disruption in the molting process of the larvae, in addition to affecting other physiological processes (Karthiga et al., 2018). AgNPs were synthesized using the leaf extract of *Mimusops elengi* in another study, and their larvicidal activity against *A. stephensi* and *Aedes albopitcus*, which are the biological vectors for malaria and arboviruses, was investigated. The authors discovered that AgNPs caused bio-toxicity by penetrating through the exoskeleton of the larvae, where they then bound with the sulfur that was present in the proteins and the phosphorus that was present in the DNA. This led to toxicity. This can lead to the denaturation of enzymes or organelles, which can further lead to a decrease in the permeability of the cellular membrane, which can cause a disturbance in proton motive force and loss of cellular function, ultimately leading to the death of the cell (Rai et al., 2009; Subramaniam et al., 2015). In general, the findings of these studies point to the conclusion that the larvicidal mechanism of AgNPs involves both physical and biochemical damage being inflicted upon the larvae. These types of damage include morphological damage, disruption of the midgut epithelium, and oxidative stress. To fully understand the larvicidal mechanism of AgNPs and to evaluate their safety and effectiveness in other organisms and the environment, however, additional research is required.

### 4.7 Antidiabetic

The human body breaks food into sugar and releases it into the bloodstream and when the level of blood sugar goes up a specific level, the pancreas releases insulin which helps in the redirection of the blood sugar into cells, when the pancreas does not produce enough insulin or the insulin produced is not getting used properly that causes accumulation of the blood sugar, which further can lead to the causation of different problems related to heart, health, etc. and this condition of improper functioning or production of insulin is designated as Diabetes. It can be further divided into 2 types, Type-1 being either autoimmune or hereditary, in this the body stops itself from producing insulin hence leading to blood sugar accumulation whereas type-2 diabetes body does not use insulin. There are different ways for the medication of type 1 and 2 diabetes. In type-1 diabetes, different types of injectable insulins’ are used, i.e. insulin, short-acting insulin, rapid-acting insulin, etc. Other than insulin Amylinomimetic injectables are also used which delay the time the stomach takes to empty itself and also reduce glucagon secretion which helps in lower blood sugar accumulation. For type 2 diabetes insulin injection and different medicine types like Alpha-glucosidase inhibitors, biguanides, etc. are used (Dirir et al., 2022).

In the recent past a variety of nanoparticles have been widely reported to exhibit antidiabetic activity by various *in vitro* and *in vivo* methods however among them, AgNPs have been most widely reported to show antidiabetic studies (Rajaram et al., 2015). According to one study, AgNPs formed from the leaf extract of *Lonicera japonica* demonstrated remarkable inhibition activity against enzymes such as alpha-glucosidase and alpha-amylase, which are responsible for high blood sugar levels by digesting starch, resulting in sugar accumulation in the blood. The enzyme inhibition was found to be reversible and non-competitive (Balan et al., 2016). AgNPs formed from the hydroethanolic extract of *Myristica fragrans* seeds having size of 50–60 nm and spherical shape also been shown to have anti-diabetic properties, as there is a noticeable inhibition of alpha-glucosidase and alpha-amylase; *M. fragrans* mediated AgNPs showed retention of glucose transport through membrane, which is observed and examined by glucose diffusion uptake assays (Perumalsamy and Krishnadhas, 2022). Nanoparticles made using *Allium cepa* having size nm and spherical shape have also shown inhibitory activity against alpha-glucosidase and alpha-amylase, hence showing antidiabetic activity (Jini and Sharmila, 2020). Additional examples of silver nanoparticles synthesized in plants that exhibit anti-diabetic characteristics can be found in Table 7.

**TABLE 7:** List of examples of plant based AgNPs with antidiabetic properties.

Plant	Shape and size of AgNPs	Activity	References
*Lonicera japonica*	Spherical and hexagonal, 53 nm	Inhibition of α amylase and α glucosidase	Balan et al. (2016)
*Myristica fragrans*	Spherical, 50–60 nm	Inhibition of α amylase and α glucosidase	Perumalsamy and Krishnadhas, (2020)
*Allium cepa*	Spherical, 49–73 nm	Inhibition of α amylase and α glucosidase	Jini and Sharmila (2020)
*Punica granatum*	Spherical, 35–60 nm	Inhibition of α amylase and α glucosidase	Saratale et al. (2018)
*Tephrosia tinctoria*	Spherical, <100 nm	Inhibition of α amylase and α glucosidase	Rajaram et al. (2015)
*Azadirachta indica*	Spherical, 34.4 nm	Pancreatic and liver cell regeneration, strong anti-diabetic potential	Rehman et al. (2023)
*Cassia auriculata*	(not specified)	Inhibition of α amylase and α glucosidase	Thirumal and Sivakumar (2021)
*Eryngium campestre* *boiss*	Spherical, 75 nm	Inhibition of α amylase and α glucosidase	Mahmoudi et al. (2021)
*Cissampelous* *Pairera*	Spherical, 84 nm	Inhibition potency against α amylase	Gauthami et al. (2021)
*Psidium Guajava*	Spherical, 52.12 nm to 65.02 nm	High antidiabetic activity	Nagaraja et al. (2022)

### 4.8 Antiangiogenesis property

The progression of cancer is accompanied by a process known as angiogenesis, in which new blood vessels are created. These newly formed blood vessels originate from pre-existing blood vessels and are involved in supplying nutrition and oxygen to the tumor and thus contribute to the development of the tumor. On the other hand, anti-angiogenesis refers to any action that inhibits the growth or construction of new blood vessels with the intention of cutting off or decreasing the blood supply to the tumor mass, which ultimately results in the suppression of the tumor. This is done in order to achieve tumor suppression. There are many different kinds of chemical-based angiogenic inhibition medicines on the market today. Some examples include Afinitor^®^, Avastin^®^, Revlimid^®^, Cometriq^®^, Inlyta^®^, Lenvima^®^, Votrient^®^, and Cyramza^®^. Other examples include these. They are employed in the treatment of cancer, despite the fact that they are associated with a wide variety of adverse effects. Some examples of these adverse effects include bleeding, clots in the arteries, hypertension, hindered wound healing, reversible posterior leukoencephalopathy syndrome (a brain disorder), and a great number of others.

**FIGURE 6:**
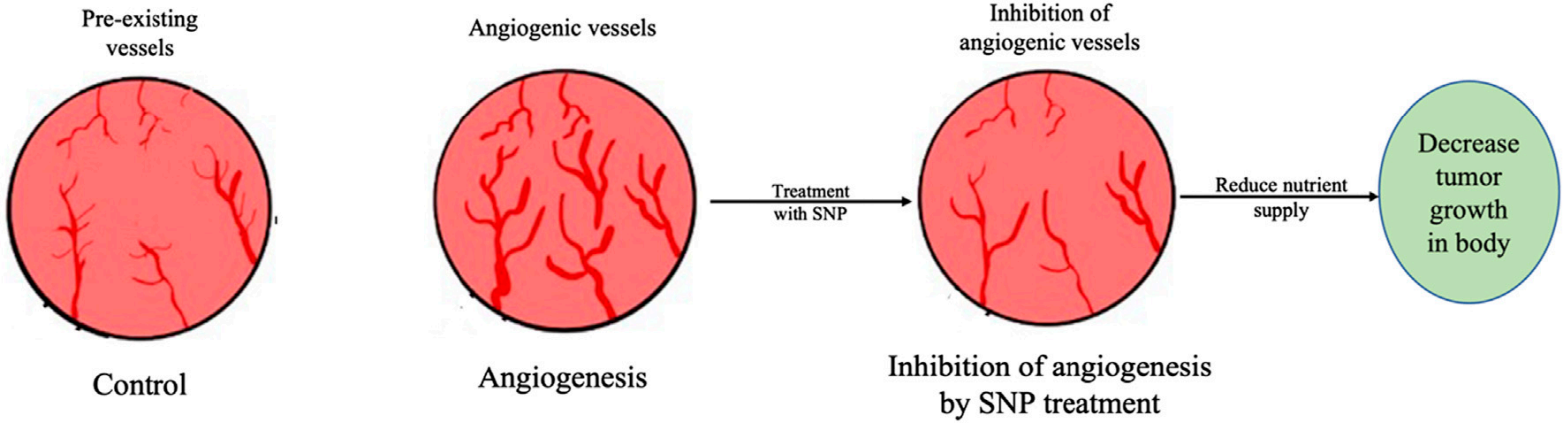
Antiangiogenic activity of silver nanoparticles synthesized using plant extracts. Antiangiogenic activity of AgNPs reduce nutrient supply to the tumor to cell by inhibiting genesis of angiogenic vessels and thus supress tumour.

AgNPs have been the subject of a significant number of research efforts in recent years, all with the objective of identifying chemical-free alternatives to already established medicinal practices. Biologically synthesized AgNPs are known to exhibit anti-angiogenic behavior, which makes them more useful in therapeutic research and less likely to be as harmful as chemical drugs. In addition, biologically synthesized AgNPs are less likely to be able to be used in humans. Historically, a number of researchers have investigated the possibility of employing it in cancer and disease treatment protocols. According to the findings of research that was carried out on the AgNPs that were synthesized from the flower extract of *Achillea biebersteinii*, which were observed to be 12 nm in size and have a spherical shape, it has demonstrated antiangiogenic properties in the aortic ring model of rats. These characteristics include having a spherical shape and having a size of 12 nm (Baharara et al., 2014). When applied to chick chorioallantoic membrane, the use of AgNPs extracted from *Salvia officinalis* demonstrated anti-angiogenic characteristics. This study showed that AgNPs have a dosage-dependent effect on the chorioallantoic membrane as on high dosage cellular extension and cell cluster formation was noticed whereas low dosage inhibited the blood vessel formation (Baharara et al., 2014). Table 8 presents a number of additional instances of antiangiogenic properties exhibited by AgNPs that have been synthesized from plant materials. Figure 6 provides a concise summary of the anti-angiogenic activity of silver nanoparticles produced through biological synthesis.

**TABLE 8:** List of examples of plant based AgNPs with angiogenic inhibitory properties.

Plant	Shape and size of AgNPs	Activity	References
*Achillea* *Biebersteinii*	Spherical, 12 nm	Angiogenic activity in rat aortic ring model	Baharara et al. (2014)
*Saliva officinalis*	Spherical, 16.5 nm	Angiogenic activity in Chick chorioalntoic membrane	Baharara et al. (2014)
*Azadirachta indica*	Spherical, 58 nm	Angiogenic activity in Chick chorioalntoic membrane	Kitimu et al. (2022)
*Praecitrullus fistulosus*	Spherical, 24 nm	CAM assay using ovo model	Madhu et al. (2022)
*Uvaria narum*	Spherical, 7.13 nm	Angiogenic activity in Chick chorioalntoic membrane	Ajaykumar et al. (2023)
*Ceropegia juncea*	Spherical, 3–32 μm	CAM assay	Subramaniam et al. (2021)
*Decalepis hamiltonii*	-	Erlich ascites murine carcinoma model	Madhu et al. (2017)
*Amaranthus cruentus*	Spherical, 15 nm	Angiogenic activity in Chick chorioallantoic membrane	Baghani and Es-haghi (2019)
*Clitoria ternatea*	Spherical, 64 nm	Microvessel density counts and CAM assays	Srinivas et al. (2019)

### 4.9 Neuroprotective property

Neurodegenerative disorders are characterized by the significant and selective loss of neurons in the motor, cognitive, and sensory systems, these disorders are chronic and progressive in nature. These conditions affect millions of people all over the world and can cause significant disability for those who suffer from these conditions (Checkoway et al., 2011). Currently, there are limited treatments for these conditions, but there is hope that new therapies may be developed in the future using plant-derived silver nanoparticles (AgNPs). Plant-derived silver nanoparticles have been found to have potential therapeutic applications in treating neurodegenerative diseases through various direct and indirect means. Currently, researchers around the world are focusing on plant-derived silver nanoparticles for the treatment of neurological disorders such as Alzheimer’s, Parkinson’s, multiple sclerosis, etc. These nanoparticles have been shown to possess unique properties that make them effective in targeting and influencing neurons by interacting with stem-cell niche pro-neurogenic factors, which could be helpful in treating neurodegenerative diseases by promoting the proliferation, differentiation, and self-renewal of neurons (Asefy et al., 2021).

According to a study, silver nanoparticles can penetrate the blood-brain barrier and target specific cells in the brain for effective drug delivery, which could be beneficial in treating these conditions by the use of a phytocarrier (Segneanu et al., 2022). According to a study, AgNPs synthesized using the aqueous extracts of *Lampranthus coccineus* and *Malephora lutea* (shape and size Spherical, 12.86–16.31 nm and Spherical, 18.66–28.19 nm respectively) were evaluated for their anti-acetylcholinesterase activity *in vivo* in mice models. The AgNPs synthesized from *L. coccineus* and *M. lutea* exhibited high anti-acetylcholinesterase activity at 0.82 ng/mL and 1.36 ng/mL respectively while the anti-acetylcholinesterase activity of synthesized AgNPs was comparable to rivastigmine, which is a standard drug used for the treatment of neurodegenerative diseases. The authors also suggested that the AgNPs upon crossing the blood-brain barrier increase acetylcholinesterase levels and decrease oxidative stress which can prove useful in the treatment of Alzheimer’s disease (Youssif et al., 2021). In a similar study AgNPs synthesized using the aqueous extract of *Aquilegia pubiflora* were studied for their anti-Alzheimer’s properties by *in vitro* assays. During the study, biologically synthesized AgNPs were found to be spherically shaped and size 19 nm exhibited the anti-Alzheimer’s properties by the inhibition of Acetylcholinesterase and Butyrylcholinesterase using Elman’s procedure in a dose-dependent manner. Biologically synthesized AgNPs inhibited the activity of Acetylcholinesterase and butyrylcholinesterase with IC50 values of 288.2 μg/mL and 269.2 μg/mL respectively (Jan et al. (2021).

Parkinson’s disease is another neurological condition that impairs balance, and coordination, and can cause trembling, stiffness, and uncontrollable movements. It can also impair speech and walking (Politis et al., 2010). The “PINK1” gene, which is typically linked to Parkinson’s disease, targets mitochondrial serine/threonine kinase, which is involved in the control of cell proliferation, differentiation, and death. An early-onset variant of autosomal recessive Parkinson’s disease is caused by mutations in the PINK1 gene (Deas et al., 2009). In a study by Shivanna et al. (2022), it was discovered that AgNPs made from *Datura stramonium* leaf extract of spherical shape and 39–59 nm size have the power to alter the regulation of the PINK1 gene. It was found that after being fed with the AgNPs, the flies had higher geotaxis behavior and a consequently favorable survival percentage in the investigation, which was conducted *in vivo* on a mutant Parkinson’s Disease model of *Drosophila melanogaster*. The scientists came to the conclusion that although additional research on molecular pathways is required to fully comprehend how AgNPs affect neurodegenerative illnesses, they have the potential to be an effective Parkinson’s disease treatment. Table 9 provides additional examples of the neuroprotective properties demonstrated by AgNPs synthesized from plant materials.

**TABLE 9:** List of examples of plant based AgNPs with neuroprotective ability.

Plant used for synthesis	Shape and size of AgNPs (nm)	Method	References
*Urtica dioica*	Spherical and oval, 29 to 70	Locomotor behavior (climbing ability) in *drosophila*, jumping ability of agnps-exposed organisms	Singh et al. (2022)
*Aquilegia pubiflora*	Spherical, 19	Acetylcholinesterase (AChE) and butyrylcholinesterase (BChE) inhibition potential by Elmasn’s procedure	Jan et al. (2021)
*Lampranthus coccineus*	Spherical, 12.86–16.31	Effect on brain glutathione (GSH) in Alzheimer-induced rats, Effect on brain malondialdehyde (MDA) in Alzheimer-induced rats, antiacetylcholinesterase activity	Youssif et al. (2019)
*Malephora lutea*	Spherical, 18.66–28.19	Effect on brain glutathione (GSH) in Alzheimer-induced rats, Effect on brain malondialdehyde (MDA) in Alzheimer-induced rats, antiacetylcholinesterase activity	Youssif et al. (2019)
*Datura stramonium*	Spherical,39–59	Geotaxis assay and PINK 1 gene expression	Shivanna et al. (2022)
*Nicotiana tabacum*	2.1	Neurotoxic shock to rat PC-12 cells	Sharma (2017)

### 4.10 Antioxidant activity

The fact that the oxidation process generates a significant amount of energy that can be used by the body does not change the fact that the oxidative stress produced by this oxidation process can cause a variety of disorders in the human body. Even though the body can use the energy that is generated by the oxidation process, this does not change the fact that the oxidation process produces oxidative stress. Free radicals, such as reactive oxygen species, are produced as a by-product of the oxidation biochemical process (ROS). Because free radicals are both unstable and reactive, they have the potential to affect the physiology of cellular macromolecules, which can lead to damage. This process is known as oxidative stress. Free radicals can cause damage to cells in a number of ways. ROS are a family of oxygen-containing molecules that are unstable and have a high propensity for interacting with other molecules found within a cell. These reactive oxygen species have the potential to accumulate inside cells, where they can cause damage to RNA, DNA, proteins, and lipids, as well as the demise of cells (Thakur et al., 2020). Antioxidants are compounds that are known to avoid oxidation through a chemical process that can result in the quenching or destruction of free radicals. Antioxidants are compounds that are known to avoid oxidation through a chemical process. Antioxidants are known to safeguard cells from damage caused by free radicals, and biologically synthesized AgNPs have a high level of antioxidant activity as they possess bioactive molecules on their surface (Zehiroglu and Ozturk Sarikaya, 2019; Keshari et al., 2020). AgNPs perform the role of electron donors, which enables them to combine with free radicals and transform them into more stable products, which in turn helps to bring an end to reactions that produce free radicals. According to the literature, the antioxidant properties of silver nanoparticles synthesized from plants can be assessed using the 2,2-diphenyl-1-picrylhydrazyl (DPPH) total antioxidant assay, ferric reducing antioxidant power (FRAP) assay, reducing power assay, 2,2′-azino-bis (3-ethylbenzothiazoline-6-sulphonic acid) radical cation (ABSTS°+) scavenging assay, hydrogen peroxide hydroxyl radical reducing power assay, and superoxide radical scavenging assay.

**FIGURE 7:**
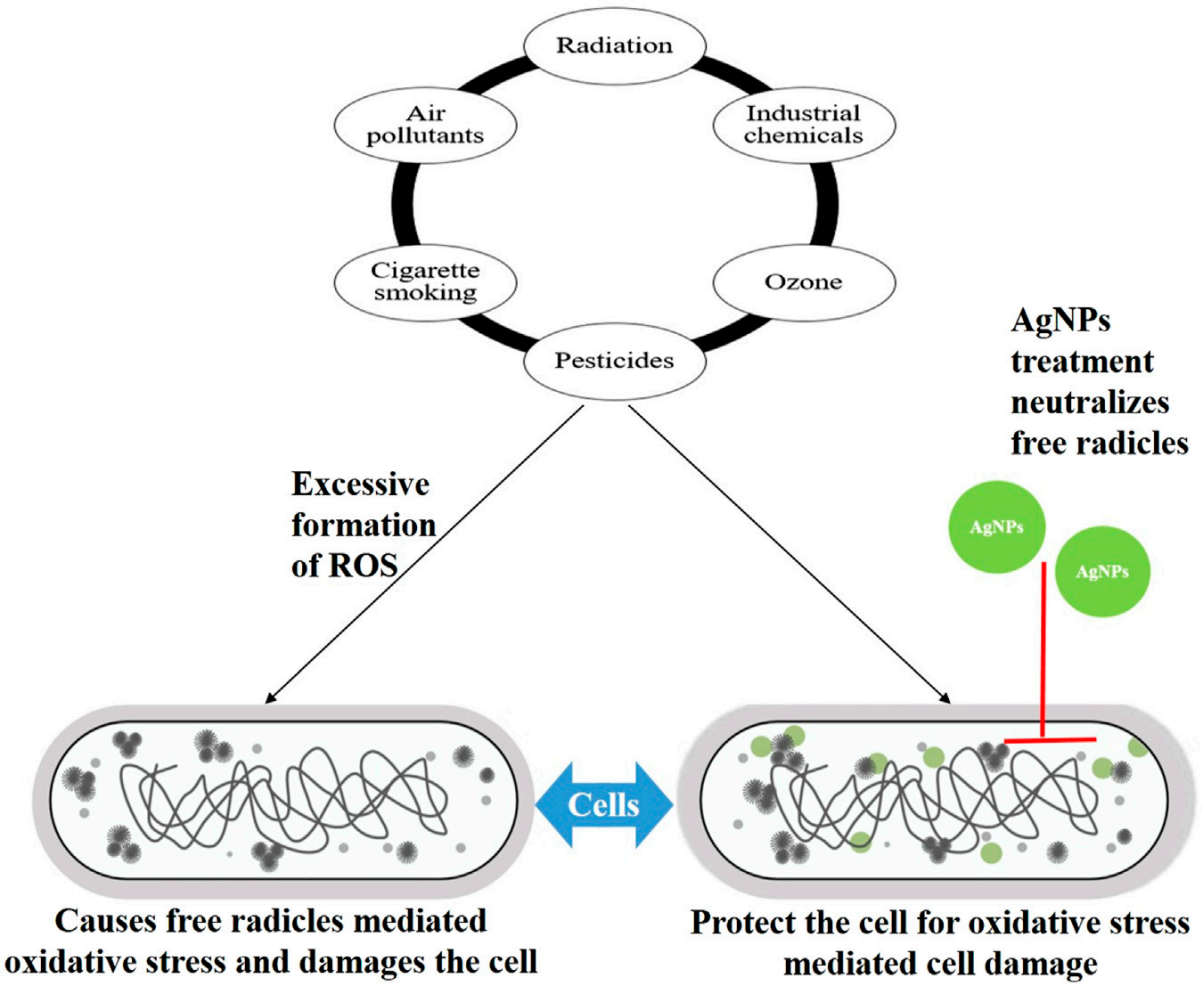
Illustration shows how silver nanoparticles synthesized using plant extracts provide protection to cells from free radicals-mediated oxidative stress. Here AgNPs: silver nanoparticles; ROS: Reactive oxygen species.

AgNPs with an average size of 34 nm and a spherical shape, which were synthesized from the leaves of *Artocarpus altilis*, exhibited antioxidant activity against DPPH free radicals that were comparable to that of ascorbic acid (Ravichandran et al., 2016). In a different piece of research, it was discovered that the spherical AgNPs with a size of 25.2 nm that were biologically synthesized from *N. sativa* could be neuroprotective agents against the oxidative stress that is the major cause of diabetic neuropathy (Alkhalaf et al., 2020). In one more study, the antioxidant property of green synthesized nanoparticles of size around 22 nm and spherical shape using plant extract of *Anagallis monelli* was demonstrated by DPPH, total antioxidant activity (TAA) assays, and the ferric reducing antioxidant power (FRAP) method (Dridi et al., 2022). In a separate piece of research, biosynthesized silver nanoparticles derived from *Echinacea purpurea* (L.) Monech demonstrated antioxidant activity in three separate assays: the ABTS assay, the DPPH scavenging assay, and the reducing power assay (Gecer et al., 2022). The antioxidant activity of silver nanoparticles created through plant extracts is presented in a condensed form in Table 10 and Figure 7.

**TABLE 10:** List of examples of plant based AgNPs with antioxidant properties.

Plant used for synthesis	Shape and size of AgNPs (nm)	Activity against	References
*Atrocarpus* *altilis*	Spherical and polydisperse, 34	DPPH	Ravichandran et al. (2016)
*Blighia sapida*	Spherical, 50–70	DPPH, reducing power assay	Akintola et al. (2020)
*Cestrum nocturnum*	Spherical, 20	DPPH, hydrogen peroxide, hydroxyl radical and superoxide radical scavenging methods	Keshari et al. (2020)
*Brassica oleracea*	Spherical, 20	DPPH, nitric oxide, hydrogen peroxide, super oxide radical scavenging	Ansar et al. (2020)
*Couroupita guianensis* *aubl*	Spherical, 4.5	DPPH	Singh et al. (2021)
*Zingiber officinale*	Spherical, 6.5	DPPH	Riaz et al. (2020)
*Helicteres isora*	Spherical, 16–95	DPPH, hydrogen peroxide scavenging assay, nitric oxide radical scavenging assay, reducing power assay	Bhakya et al. (2015)
*Bergenia ciliata*	Spherical, 35	DPPH, total antioxidant assay	Phull et al. (2016)
*Piper longum*	Spherical, 46	DPPH, NO radical scavenging, superoxide scavenging	Reddy et al. (2014)
*Elephantopus scaber*	Spherical, 78	DPPH	Kharat and Mendulkar (2016)

### 4.11 Antiplasmodial activity

*Plasmodium* sp. can cause an acute febrile illness known as malaria and malaria can spread to humans through the bite of infected female *Anopheles* mosquitoes. Nearly one-half of the total world’s population was at risk of malaria in 2021. In the 21st century, tremendous efforts by the WHO in reducing the infection rate by controlling vectors and using preventive synthetic antimalarial drugs have shown progress in controlling malaria worldwide but also given rise to another problem of emerging resistance towards insecticides among *Anopheles* sp. mosquitoes (WHO, 2023). There are plenty of antimalarial drugs available in the market however, these drugs come with plenty of side effects like gastrointestinal problems (Mild nausea or diarrhea) or mucocutaneous problems such as reversible skin or mucosal pigmentation (Kukde et al., 2023). According to the findings of one of the studies, AgNPs with a size of 41 nm and a spherical shape were biologically synthesized from an aqueous leaf extract of *S. officinalis*. These AgNPs demonstrated high antiplasmodial activity against *Plasmodium falciparum* 3D7, which suggests that they may be a viable alternative to the synthetic antimalarial drugs currently in use (Okaiyeto et al., 2020). Biologically synthesized AgNPs using the leaf extract of Alchornea cordifolia were of size 11.77 ± 5.57 nm, spheroidal, and polycrystalline, and they showed high levels of antiplasmodial activity against 3D7 and RKL9 Plasmodium falciparum strains. This was due to their small size and high surface area to volume ratio, which facilitates their potential to penetrate cell membranes (Kojom Foko et al., 2023). According to the findings of one study, the crude extracts of *Azadirachta indica* exhibited dose-dependent antiplasmodial activity (Hawadak et al., 2022). The precise mechanism by which AgNPs exert their antiplasmodial activity is not completely understood and will be the focus of researchers’ investigations in the years to come. Table 11 contains even more instances of AgNPs exhibiting antiplasmodial activity than those described above.

**TABLE 11:** List of examples of plant based AgNPs with anti plasmodial properties.

Plant	Shape and size of AgNPs (nm)	Activity against	References
*Azadirchata* *indica*	Spheroidal, crystalline, 13–19.30	3D7 and RKL9 *Plasmodium falciparum* strains	Hawadak et al. (2022)
*Azadirchata* *indica* *and* *Ocimum sanctum*	Spherical, 4.74–39.32	*Plasmodium falciparum* 3D7	Sardana et al. (2018)
*Catharanthus roseus*	Spherical, 1–10	*Plasmodium falciparum* 3D7	Ponarulselvam et al. (2012)
*Salvia officinalis*	Spherical, 41	*Plasmodium falciparum* 3D7	Okaiyeto et al. (2022)
Alchornea cordifolia	Spheroidal, and polycrystalline, 11.77 ± 5.57	*Plasmodium falciparum* 3D7 and RKL9	Kojom Foko et al. (2023)
*Azadirchata* *indica*	Spherical shape, 2–8	*Plasmodium falciparum* in human blood cell culture	Mishra et al. (2013)
*Saraca asoca*	Spherical shape, 5–20	*Plasmodium falciparum* in human blood cell culture	Mishra et al. (2013)
*Pteridium aquilinum*	Spherical, 35–65	CQ-resistant (CQ-r) and CQ-sensitive (CQ-s) strains of Plasmodium falciparum	Panneerselvam et al. (2016)
*Crataegus ambigua*	Spherical, 32	*Plasmodium falciparum* NF54 strain	Ojemaye et al. (2021)
*Andrographis paniculata* Nees	Spherical*, 35–55*	*Plasmodium falciparum*	Panneerselvam et al. (2011)

### 4.12 Toxicity, ecotoxicity, and biocompatibility of plant-mediated silver nanoparticles (AgNPs)

It is imperative to assess the potential environmental impact and effects on human health by examining the toxicity, ecotoxicity, and biocompatibility of silver nanoparticles (AgNPs) synthesized through plant-mediated processes. This extensive analysis will delve into the detrimental impacts of AgNPs on living organisms, ecosystems, and their interaction with biological systems.

Numerous studies have investigated the potential toxicity of AgNPs across various biological systems, including human cells and animal models. The correlation between the small size and heightened surface area of AgNPs enhances their reactivity, fostering interactions with biological components. Cellular uptake of AgNPs has been shown to induce oxidative stress, resulting in DNA damage, protein denaturation, and lipid peroxidation. Additionally, AgNPs exhibit pathogenic potential by disrupting cellular processes and activating apoptotic pathways, as highlighted in Table 12.

**TABLE 12:** Effect of AgNPs on different human cells.

Cell	Shape and size of AgNPs	Action	References
Lymphocytes	Spherical, 150–200 nm	AgNPs ≤20 nm - cell shrink	Zhornik et al. (2015)
		AgNPs ≥200 nm- no effect	
Alveolar epithelial cells	Spheres = 30 nm; wires = length: 1.5–25 μm	sphere = no effect	Stoehr et al. (2011)
		wires = increase LDH release from cells	
Skin keratinocytes	Spherical, 9.1 ± 2.6 nm	3D EpiKutis model = cell viability was greater than 80%	Chen et al. (2019)
		2D keratinocytes = cell viability 11%	
Human epidermal keratinocytes	Spherical, 5 and 40 nm	Non-cytotoxic concentrations of AgNPs 25 μg/mL	Galandakova et al. (2016)
Neuroblastoma cells	Spherical, 5–75 nm	Size-dependent toxicity on SH-SY5Y cells	Zhai et al. (2022)

Concerns arise regarding the ecotoxicological impact of AgNPs released into aquatic habitats, whether through direct disposal or runoff from treated surfaces. Aquatic organisms, including algae, crustaceans, and fish, may face exposure to AgNPs, leading to adverse effects on their internal physiology and behavior. Toxic effects on aquatic biota include reduced reproduction, altered development, and disruptions in metabolic pathways, with potential long-term consequences arising from AgNP accumulation in sediments.

While certain circumstances may pose risks, the biocompatibility of AgNPs is dictated by factors such as size, shape, and surface functionalization. Plant-mediated synthesis often yields less harmful, stabilized AgNPs. Research demonstrates the utility of biocompatible AgNPs in biomedical applications, such as imaging and drug delivery. Controlled release in specific biological contexts enhances therapeutic efficacy while minimizing adverse effects on healthy tissues.

To mitigate potential adverse effects, researchers are exploring various approaches, including surface modification and encapsulation, to enhance biocompatibility and reduce the release of hazardous ions. Understanding factors influencing AgNP toxicity, including concentration and exposure duration, is crucial for establishing safe usage guidelines. A comprehensive understanding of the toxicity, ecotoxicity, and biocompatibility of plant-mediated AgNPs is essential as their utilization expands. Advances in nanotoxicology and ecotoxicological assessments will contribute to developing recommendations for the safe and sustainable use of plant-mediated AgNPs across diverse applications.

## 5 Conclusion

This research conducts a comprehensive exploration of silver nanoparticles (AgNPs) synthesis and characterization, focusing on the environmentally friendly and cost-effective biological approach using plant extracts. Comparative assessment with traditional methods highlights the environmental advantages and ease of production in biological synthesis. The review underscores the diverse medicinal properties of plant-derived AgNPs, including antibacterial, anti-biofilm, cytotoxic, wound healing, bone healing, larvicidal, anti-diabetic, anti-angiogenic, antioxidant, and antiplasmodial activities. Despite acknowledged therapeutic potential, a substantial gap exists in realizing widespread nanoparticle applications in healthcare. Closing this gap necessitates rigorous research into more effective biomolecules for silver ion reduction and developing stable AgNPs for commercial utilization, promising advancements in human health and medicine.
